# Supplementary material for: Sodium, Potassium and Iodine Intake in an Adult Population of Lithuania
Source: Nutrients. 2022 Sep 16;14(18):3817. doi: 10.3390/nu14183817 (PMC9504939; doi:10.3390/nu14183817)
Supplement: Supplementary file 1 [file nutrients-14-03817-s001.zip › nutrients-1887398-supplementary.pdf]

„Lietuvos gyventojų natrio ir jodo būklės vertinimas ir visuomenės sveikatos politikos gairių sudarymas (NATRIJOD)“

DRUSKOS VARTOJIMO ĮPROČIŲ IR SVEIKATOS BŪKLĖS KLAUSIMYNAS

Dėkojame, kad sutikote dalyvauti Lietuvos ir Pasaulio sveikatos organizacijos tyrėjų atliekamame tyrime. Maloniai prašome atidžiai perskaityti kiekvieną klausimą ir tinkamą atsakymą pažymėti kryželiu („X“), ar įrašyti žodžiu. Apklausa yra anoniminė, konfidencialumas garantuojamas, gauti duomenys bus naudojami tik apibendrintai. Jūsų atsakymai, nuomonė ir pasiūlymai yra labai svarbūs vertinant Lietuvos gyventojų kasdienius druskos vartojimo įpročius ir jų pokyčius bei teikiant rekomendacijas valstybės institucijoms ir privačiam maisto gaminimo sektoriui kaip sumažinti druskos vartojimą ir pagerinti kiekvieno gyventojo sveikatos būklę, darbingumą ir kartu visuomenės sveikatą.

**TIRIAMOJO NUMERIS** (*pildo tyrėjas - vieta etiketei užklijuoti*)<br>Tyrėjas	
**Bendroji informacija**	
Klausimyno pildymo data (metai/ mėnuo/diena)	
Sutikimo forma perskaityta ir pasirašyta	☐ Taip ☐ Ne *(jei ne, dalyvauti negalima)*
**24 val. šlapimo mėginio rinkimas**	
Rinkimo pradžios data (metai/mėnuo/diena)	
Rinkimo pradžios laikas (val./min)	
Rinkimo pabaigos data (metai/mėnuo/diena)	
Rinkimo pabaigos laikas (val./min)	
Surinkto šlapimo tūris (ml)	
**Fiziniai duomenys *(pildo tyrėjas)***	
Ūgis (cm) *(be batų)* [aparato tipas]	
Svoris (kg) *(be batų, vilkint lengvus drabužius)* [aparato tipas]	
Arterinis kraujo spaudimas (mmHg) [aparato tipas, manžetės dydis]	AKS 1 matavimas ..../................<br>AKS 2 matavimas ..../................<br>AKS 3 matavimas ..../................
Širdies susitraukimų dažnis (pulsas) k/min	ŠSD 1 matavimas ....<br>ŠSD 2 matavimas ....<br>ŠSD 3 matavimas ....

**Demografinė informacija**		
1. Jūsų lytis	☐ Moteris	☐ Vyras
2. Jūsų gimimo data		
3. Gyvenamoji vieta	☐ Kaimas ☐ Miestas	
4. Jūsų išsilavinimas (baigti mokslai)	☐ Pagrindinis ☐ Vidurinis ☐ Specialusis vidurinis<br>☐ Aukštesnysis (technikumas, aukštesnioji mokykla)	

	☐ Aukštasis koleginis ☐ Aukštasis universitetinis
5. Šeiminė padėtis	☐ Vedęs (ištekėjusi) arba gyvenate nesusituokęs (-usi)<br>☐ Nevedęs (netekėjusi) ☐Išsiskyręs (-usi) ☐Našlys (-ė)<br>____
6. Jūsų pagrindinis užsiėmimas pastaruosius 12 mėn.	☐ Dirbantis ☐ Studentas, (–ė) ☐ Bedarbis, ☐ Pensininkas,(–ė) ☐ Kita (įrašykite)__________________

Druskos vartojimo įpročiai

7. Ar sūdote valgydami jau pagamintus patiekalus?	☐ Niekada<br>☐ Truputį, jei maistas nepakankamai sūrus<br>☐ Beveik visada, net neragaudamas<br>☐ Visada
8. Kaip dažnai naudojate druską gamindami maistą namuose?	☐ Niekada ☐ Kartais ☐ Dažnai<br>☐ Visada
9. Kokios rūšies druską vartojate maistui gaminti ar sūdyti?	☐ Joduotą druską (akmens, jūros ar kitą)<br>☐ Nejoduotą akmens druską<br>☐ Himalajų druską<br>☐ Kita (įrašykite)___________________________<br>☐ Nežinau
10. Kaip dažnai valgiui gaminti ar sūdyti naudojate joduotą druską?	☐ Niekada ☐ Kartais ☐ Dažnai<br>☐ Visada
11. Jei nenaudojate joduotos druskos, nurodykite kodėl?	☐ Turiu per mažai informacijos apie joduotą druską<br>☐ Esu įpratęs (-usi) naudoti nejoduotą druską<br>☐ Manau, kad pakankamai jodo gaunu su kitu maistu<br>☐ Rinkdamasis (-i) druską, nesidomiu, ar joje yra jodo<br>☐ Nežinau<br>☐ ☐ Kita (*įrašykite*)
12. Ar joduotą druską laikote sandariame, užsuktame inde?	☐ Taip, joduotą druską laikau sandariame inde<br>☐ Ne, laikau atvirai<br>☐ Tokios druskos nenaudoju
13. Kada atidarėte pastarąją joduotos druskos pakuotę?	☐ prieš 0–3 mėn. ☐ prieš 4–6 mėn.<br>☐ prieš 6 mėn. ir anksčiau ☐ nežinau<br>☐ joduotos druskos nevartoju
14. Ar naudojate jodo turinčius maisto papildus, jūros dumblius ar spiruliną?	☐ Taip ☐ Ne ☐ Nežinau

15. Kuriuos maisto produktus dažniausia sūdote gamindami maistą ar valgydami namuose paruoštą maistą? Pažymėkite „X“. Jei niekada nevartojate paminėto maisto – pažymėkite „Netaikoma“

	Papildomai nesūdau	Sūdau gamindamas (-a)	Sūdau jau pagamintą patiekalą	Nevartoju šio produkto
Virtos bulvės	☐	☐	☐	☐
Keptos bulvės	☐	☐	☐	☐
Makaronai arba ryžiai	☐	☐	☐	☐
Kepti kiaušiniai	☐	☐	☐	☐
Virti kiaušiniai	☐	☐	☐	☐
Šviežios daržovės	☐	☐	☐	☐
Virtos daržovės	☐	☐	☐	☐
Konservuotos daržovės	☐	☐	☐	☐

Troškintos daržovės	☐	☐	☐	☐
Žalia mėsa *(pvz., kepsnys, mėsos filė)*	☐	☐	☐	☐
Perdirbta mėsa *(pvz., šnicelis, marinuota mėsa)*	☐	☐	☐	☐
Šviežia, šaldyta žuvis *(pvz., filė)*	☐	☐	☐	☐
Perdirbta žuvis *(pvz., žuvų piršteliai)*	☐	☐	☐	☐

16. Kaip dažnai valgote šiuos sūdytus maisto produktus?

	Niekada	Rečiau nei vieną kartą per savaitę	1–2 kartus per savaitę	3–5 kartus per savaitę	Vieną kartą per dieną	Kelis kartus per dieną
Bulvių ar kukurūzų traškučius (čipsus)	☐	☐	☐	☐	☐	☐
Sūdytus ar skrudintis riešutus, sūdytus kukurūzų spragėsius	☐	☐	☐	☐	☐	☐
Sūdytą, vytintą ar rūkytą žuvį, silkę, šprotus, žuvies konservus	☐	☐	☐	☐	☐	☐
Rūkytus, sūdytus ar vytintus mėsos gaminius (*pvz., rūkytas dešras, kumpius*)?	☐	☐	☐	☐	☐	☐
Greitai paruošiamas užpilamas sriubas, sultinio kubelius	☐	☐	☐	☐	☐	☐

17. Kaip manote, kiek druskos Jūs suvartojate?	☐ Gerokai per daug ☐ Per daug<br>☐ Tinkamą kiekį<br>☐ Per mažai ☐ Gerokai per mažai ☐ Neturiu nuomonės
18. Kiek gramų druskos rekomenduojama suvartoti suaugusiam žmogui per parą? *(pasirinkite vieną atsakymo variantą)*	☐ Iki 5 g (nepilnas arbatinis šaukštelis)<br>☐ 5–10 g (iki 1 arbatinio šaukštelio su kaupu)<br>☐ 10–15 g<br>☐ Daugiau nei 15 g<br>☐ Nežinau
19. Kaip manote, ar didelis druskos suvartojimas gali sukelti rimtų sveikatos sutrikimų?	☐ Taip ☐ Ne ☐ Nežinau
20. Ar imatės priemonių suvartojamam druskos kiekiui reguliuoti? *(nurodykite tiek atsakymų, kiek Jums atrodo tinkama)*	☐ Taip ☐ Ne<br><br>Jei TAIP, kokias priemones ar būdus naudojate?:<br>☐ Vartoju kiek įmanoma mažiau perdirbto maisto, pusgaminių<br>☐ Renkuosi simboliu „Rakto skylute“ pažymėtus produktus<br>☐ Perskaitau maisto produktų etiketes apie druskos ar natrio kiekį juose<br>☐ Valgydamas/-a papildomai druskos nededu<br>☐ Perku maisto produktus, turinčius mažai druskos ar natrio *(pvz., silpnai sūdytus, be druskos)*<br>☐ Gamindamas/-a maistą druskos nededu<br>☐ Druską keičiu prieskoniais be druskos<br>☐ Vengiu valgyti viešojo maitinimo įstaigose

	☐ Kita (išvardinkite):________________________________ _
21. Kur, Jūsų nuomone, tikslinga sumažinti druskos kiekį? *(nurodykite tiek atsakymų, kiek Jums atrodo tinkama)*	☐ Duonoje<br>☐ Padažuose (pvz., majoneze, sojos padaže)<br>☐ Sūdytoje, vytintoje ar rūkytoje žuvyje, silkėje<br>☐ Kulinarijos skyriuose parduodamuose keptuose mėsos ir žuvies gaminiuose (pvz., keptuose viščiukų ketvirčiuose, kiaulienos karbonaduose, kepsneliuose)<br>☐ Kulinarijos skyriuose parduodamuose keptuose bulvių gaminiuose (pvz., plokštainyje, blynuose, vėdaruose)<br>☐ Mėsos gaminiuose (pvz., rūkytose, virtose, vytintose dešrose, dešrelėse, kumpiuose)<br>☐ Sūriuose<br>☐ Parduodamose salotose ir mišrainėse<br>☐ Viešojo maitinimo įstaigų patiekaluose<br>☐ Neturiu nuomonės

Mitybos įpročiai

22. Kaip dažnai valgote ne namuose gamintą maistą?

	Niekada	Rečiau nei 1 kartą per savaitę	1–2 kartus per savaitę	3–5 kartus per savaitę	Kasdien	Kelis kartus per dieną
Viešojo maitinimo įstaigose	☐	☐	☐	☐	☐	☐
Paruoštą vartoti maistą, įsigytą parduotuvėje ar viešojo maitinimo įstaigoje *(pvz., sumuštiniai, salotos, kepta mėsa, vyniotiniai, pica)*	☐	☐	☐	☐	☐	☐
Parduotuvėje pirktus pusgaminius *(pvz., koldūnus, picą, marinuotą mėsą, šviežias dešreles)*	☐	☐	☐	☐	☐	☐

23. Kiek vandens išgeriate per dieną?	Stiklinės/-ių (įprasta stiklinė 240 ml)___________
24. Kiek kitų skysčių (išskyrus vandenį) suvartojate per dieną?	Stiklinės/-ių ________________________________
25. Kiek arbatos ir kavos išgeriate per dieną?	Puodeliai ________________________________

26. Kaip dažnai valgote vaisius ir daržoves *(išskyrus bulves)*?

	Nevartoju	Rečiau nei vieną kartą per savaitę	1–2 kartus per savaitę	3–5 kartus per savaitę	6–7 kartus per savaitę	Kelis kartus per dieną
Kaip dažnai valgote vaisių? *(pvz., bananai, slyvos, abrikosai, nektarinai, vynuogės, apelsinai, kiviai, avokadas, kriaušės, vyšnios, braškės)*	☐	☐	☐	☐	☐	☐
Kaip dažnai valgote džiovintus vaisius ir						

riešutus? *(pvz., džiovintos slyvos, abrikosai, žemės riešutai, migdolai)*	☐	☐	☐	☐	☐	☐
Kaip dažnai valgote šviežių daržovių? *(pvz., lapinės daržovės, morkos, kopūstai, špinatai, ropės, pomidorai, burokėliai, cukinijos)*	☐	☐	☐	☐	☐	☐
Kaip dažnai valgote virtų ar troškintų daržovių? *(pvz., lapinės daržovės, morkos, kopūstai, špinatai, ropės, pomidorai, burokėliai, cukinijos)*	☐	☐	☐	☐	☐	☐
Kaip dažnai valgote marinuotas ar raugintas daržoves?	☐	☐	☐	☐	☐	☐

27. Tomis dienomis, kai vartojate vaisių, kiek porcijų vaisių suvalgote per dieną?	Porcijų skaičius: ___________<br>*(1 porcija – vienas vidutinio obuolio dydžio vaisius)*<br>☐ Nežinau
28. Tomis dienomis, kai valgote daržovių, kiek porcijų daržovių suvalgote per dieną?	Porcijų skaičius: ______________<br>*(1 porcija šviežių daržovių atitinka vieną 240 ml stiklinę, virtų daržovių – pusė stiklinės)*<br>☐ Nežinau

29. Kaip dažnai valgote šiuos maisto produktus?

	Nevartoju	Rečiau nei vieną kartą per savaitę	1–2 kartus per savaitę	3–5 kartus per savaitę	6–7 kartus per savaitę	Kelis kartus per dieną
Pienas	☐	☐	☐	☐	☐	☐
Sūris *(pvz., pelėsinis, lydytas, rūkytas, fermentinis)*	☐	☐	☐	☐	☐	☐
Žuvis ar jūros gėrybės	☐	☐	☐	☐	☐	☐
Mėsa	☐	☐	☐	☐	☐	☐
Duona (juoda, balta), batonas	☐	☐	☐	☐	☐	☐
Sūrokas natūralus mineralinis ar šaltinio vanduo, (pvz., „Vytautas“, „Darida“, „Borjomi“)	☐	☐	☐	☐	☐	☐

30. Kiek duonos riekių ar duonos pakaitalų vidutiniškai suvalgote per dieną? *(pvz., duonos pakaitalai - džiūvėsiai, krekeriai, duonos trapučiai arba traškučiai, bet ne dribsniai)*	Juodos duonos riekių skaičius (*vnt.*)_________<br>Baltos duonos riekių skaičius (*vnt.*)_________<br>Batono riekių skaičius (*vnt.*)___________<br>Duonos pakaitalų riekių skaičius (vnt.)___________
31. Ar valgote namuose keptą duoną?	☐ Taip ☐ Ne<br>Jei TAIP, ar jai kepti naudojate joduotą druską<br>☐ Taip, naudoju joduotą druską ☐ Nenaudoju<br>☐ Nežinau
**Sveikatos būklė**	

32. Nurodykite ligas, kuriomis šiuo metu sergate ar esate persirgęs ir jų diagnozę patvirtino gydytojas ar kitas sveikatos priežiūros darbuotojas?

	Sergu ar esu persirgęs šiomis ligomis	Nesergu/ nesirgau	Susirgau per pastaruosius 12 mėn.	Sergu / ar persirgau seniau nei 1 m.

Padidėjęs kraujospūdis (arterinė hipertenzija)	☐	☐	☐	☐
Miokardo infarktas	☐	☐	☐	☐
Sutrikęs širdies ritmas (prieširdžių virpėjimas)	☐	☐	☐	☐
Širdies nepakankamumas	☐	☐	☐	☐
Ūminis galvos smegenų kraujotakos sutrikimas (insultas)	☐	☐	☐	☐
Inkstų ligos	☐	☐	☐	☐
Cukrinis diabetas	☐	☐	☐	☐
Skydliaukės veiklos sutrikimas (hipotireozė, hipertireozė), dėl kurių reikia vartoti vaistus	☐	☐	☐	☐
Kita (nurodykite)	☐	☐	☐	☐

33. Ar vartojate vaistus širdies ritmo sutrikimui gydyti?	☐ Taip ☐ Ne<br>Jei TAIP, kokius vaistus vartojate:<br>☐ kordaroną ☐ ritmonormą<br>☐ kita (įrašykite) ____________________
34. Ar pastarąsias 7 dienas vartojote vaistus skydliaukės ligai gydyti?	☐ Taip ☐ Ne<br>Jei TAIP – kokius vaistus vartojate?<br>☐ L-tiroksiną ☐ metizolį, tirozolį ar propicilį<br>☐ Kita (*nurodykite pavadinimus*)
35. Ar per pastarąsias 7 dienas vartojote kokius nors receptinius ar nereceptinius vaistus, vaistų (tinktūras) gerklės skausmui mažinti ar atsikosėjimui gerinti, maisto papildus (pvz., mineralines medžiagas, amino rūgščių preparatus, vitaminus)?	☐ Taip ☐ Ne<br>Jei TAIP, užrašykite pilną pavadinimą (-us), nurodytą (-us) ant pakuotės ir kokį kiekį (pvz., tablečių skaičių) vartojate per dieną?
**Tik MOTERIMS**<br>36. Ar šiuo metu laukiatės?	☐ Taip ☐ Ne<br>Jei laukiatės – kelinta nėštumo savaitė?<br>Ar laukiatės dvynukų ar trynukų? ☐Taip ☐ Ne<br>Ar pastojote natūraliai ☐ Taip ☐ Ne<br>Ar buvo daryta stimuliacija ☐ Taip ☐ Ne<br>Ar buvo taikyta dirbtinio apvaisinimo procedūra ☐ Taip ☐ Ne __________
**Kiti klausimai**	
37. Kiek kartų per savaitę mažiausiai 30 min. užsiimate energinga fizine veikla (mankštinatės, sportuojate, bėgiojate, greitai einate, važiuojate dviračiu, energingai dirbate namų ūkio darbus ir pan.) taip, kad pagreitėtų kvėpavimas ir pulsas? (30 min. trukmė vienos paros metu gali susidėti iš kelių dalių, pvz., 15+15 min.)	☐Neužsiimu tokia fizine veikla<br>☐ 1 dieną<br>☐ 2 dienas<br>☐ 3 dienas<br>☐ 4 dienas<br>☐ 5 dienas<br>☐ 6 dienas<br>☐ 7 dienas

38. Ar per dieną surūkote nors vieną ar daugiau cigarečių/ cigarų / pypkių?	☐ Ne, aš niekada nerūkiau<br>☐ Ne, tačiau rūkiau praeityje<br>☐ Taip, rūkau paprastas<br>☐ Taip, rūkau elektronines

Nuoširdžiai dėkojame už Jūsų skirtą laiką, atsakymus ir kantrybę pildant klausimyną.
Tikime, kad dalyvavimas šiame tyrime padės geriau suprasti savo druskos vartojimo įpročius ir atkreipti dėmesį į galimus rizikos veiksnius ir svarbius savirūpos klausimus.

Prašome kartu su užpildytu klausimynu atnešti šlapimo, surinkto pagal pateiktas rekomendacijas, indą

Linkime sveikatos!
